# An enhanced genetic mutation-based model for predicting the efficacy of immune checkpoint inhibitors in patients with melanoma

**DOI:** 10.3389/fonc.2022.1077477

**Published:** 2023-01-17

**Authors:** Chaohu Pan, Hongzhen Tang, Wei Wang, Dongfang Wu, Haitao Luo, Libin Xu, Xue-Jia Lin

**Affiliations:** ^1^ The First Affiliated Hospital, Jinan University, Guangzhou, Guangdong, China; ^2^ Zhuhai Institute of Translational Medicine, Zhuhai People’s Hospital (Zhuhai Hospital Affiliated with Jinan University), Jinan University, Zhuhai, Guangdong, China; ^3^ The Biomedical Translational Research Institute, Faculty of Medical Science, Jinan University, Guangzhou, Guangdong, China; ^4^ Department of Medicine, YuceBio Technology Co., Ltd, Shenzhen, Guangdong, China; ^5^ Department of Orthopedic Surgery, National Cancer Center/National Clinical Research Center for Cancer/Cancer Hospital, Chinese Academy of Medical Sciences and Peking Union Medical College, Beijing, China

**Keywords:** melanoma, immune checkpoint inhibitors, genetic mutation, durable clinical benefit model, machine learning classifiers

## Abstract

**Background:**

Programmed death ligand 1 (PD-L1) and tumor mutation burden (TMB) have been developed as biomarkers for the treatment of immune checkpoint inhibitors (ICIs). However, some patients who are TMB-high or PD-L1-high remained resistant to ICIs therapy. Therefore, a more clinically applicable and effective model for predicting the efficacy of ICIs is urgently needed.

**Methods:**

In this study, genomic data for 466 patients with melanoma treated with ICIs from seven independent cohorts were collected and used as training and validation cohorts (training cohort n = 300, validation cohort1 n = 61, validation cohort2 n = 105). Ten machine learning classifiers, including Random Forest classifier, Stochastic Gradient Descent (SGD) classifier and Linear Support Vector Classifier (SVC), were subsequently evaluated.

**Results:**

The Linear SVC with a 186-gene mutation-based set was screened to construct the durable clinical benefit (DCB) model. Patients predicted to have DCB (pDCB) were associated with a better response to the treatment of ICIs in the validation cohort1 (AUC=0.838) and cohort2 (AUC=0.993). Compared with TMB and other reported genetic mutation-based signatures, the DCB model showed greater predictive power. Furthermore, we explored the genomic features in determining the benefits of ICIs treatment and found that patients with pDCB were associated with higher tumor immunogenicity.

**Conclusion:**

The DCB model constructed in this study can effectively predict the efficacy of ICIs treatment in patients with melanoma, which will be helpful for clinical decision-making.

## 1 Introduction

Melanoma is a highly malignant neoplasm derived from melanocytes, which mostly occurs in the skin and accounts for approximately 3% of all tumors (1). The traditional treatments for patients with melanoma include surgical resection, chemotherapy, radiotherapy, and targeted therapy (2, 3). However, the efficacy of treatment remains limited in patients, particularly those in advanced stages. In recent years, immune checkpoint inhibitors (ICIs) targeting the programmed death-ligand 1 (PD-L1), cytotoxic T-lymphocyte antigen 4 (CTLA-4), and programmed cell death receptor 1 (PD-1) have revolutionized the treatment landscape for melanoma (4–7). These ICIs therapies can relieve immune suppression and activate T cells and other lymphocytes, allowing the immune system to attack and kill melanoma (8). However, the clinical benefit of ICIs treatment remains limited to a subset of patients, and some patients even experience severe side effects, leading to treatment discontinuation (9–12). Therefore, the development of predictive biomarkers to distinguish the responders from the non-responders is urgently needed.

PD-L1 expression and tumor mutational burden (TMB) have been confirmed by multiple clinical trials to predict the efficacy of ICIs in melanoma (13–18). However, PD-L1 expression or TMB alone was not effective enough to precisely identify responders. Some patients with PD-L1-high or TMB-high remained resistant to the treatment of ICIs (19, 20); Furthermore, there is no standardized cut-off value for the PD-L1 expression or TMB (21–23). These limited the clinical application of PD-L1 expression and TMB, highlighting the importance of developing a more effective predictive biomarker.

Previous studies have constructed the genetic mutation-based signatures to predict the response to the treatment of ICIs (24–26). However, the genetic mutation-based signatures constructed by Jiang et al. and Lu et al. did not strictly screen the samples, resulting in the inclusion of the post treatment samples (24, 25). On the other hand, the genetic mutation-based signature constructed by Long et al. was based on the panel sequencing, and its predictive ability for melanoma can be further improved (26).

Based on the whole exome sequencing (WES) and clinical data of patients with melanoma collected from the pre-treatment with ICIs, we aimed to construct a durable clinical benefit (DCB) model to predict the response to ICIs in patients with melanoma. The prediction capabilities of TMB and other reported genetic mutation signatures were also evaluated and compared with our DCB model.

## 2 Materials and methods

### 2.1 Study cohorts

Clinical and WES data of 644 patients with melanoma treated with ICIs were obtained from seven cohorts (15, 27–32). A cohort of patients with melanoma who only received the treatment of ICIs were obtained based on whether they had received prior ICIs therapy and combination therapy, such as ICIs combined with chemotherapy or targeted therapy. After determining the time point at which the biopsy was obtained and selecting patients for imaging evaluation, a final cohort of 466 patients with melanoma were eventually established (**Table S1**). The training cohort (n = 300) consisted of Hugo, Riaz, Nathanson, Liu and Miao cohorts; validation cohort1 (n = 61) and 2 (n =105) were the Snyder cohort and Allen cohort, respectively. The DCB was defined as complete response (CR), partial response (PR), or stable disease (SD) with progression-free survival (PFS) more than 24 weeks. No durable clinical benefit (NDB) was defined as progressive disease (PD) or SD with a PFS less than 24 weeks.

In addition, clinical and genomic data for 202 patients with lung cancer and 261 patients with clear cell renal cell carcinoma (ccRCC) treated with ICIs, as well as 287 patients with melanoma without receiving ICIs therapy (TCGA-skcm_2015), were obtained from published literature (33–36) and cbioportal (https://www.cbioportal.org/datasets), respectively, to further evaluate the DCB model.

The format of all mutation data in this study was mutation annotation format. Non-synonymous mutations were retained, and the data were transformed from the mutation annotation format to the sample gene matrix. For genes with at least one mutation, the mutation status was classified as mutation.

### 2.2 Construction and validation of the DCB model

Ten classifiers, namely BernoulliNB, ComplementNB, Linear SVC, Adaptive Boosting (AdaBoost), stochastic gradient descent (SGD), Gradient Boosting, Extra Trees, Random Forest, Decision Tree and Extra Tree were used to construct the DCB model to predict the response of patients with melanoma treated with ICIs in the training cohort (Python ‘scipy’ and ‘sklearn’ packages). First, the recursive feature elimination method was used to rank features by importance. The iteration starts with the most important features. One feature was added at each iteration, and the f1 scores of fitting and generalization, as well as the sum of the scores, were calculated. The iteration was stopped when the maximum value of the sum of the scores did not change for the next 100 iterations. These operations were performed for each classifier to obtain the maximum sum of the scores. Subsequently, the maximum sum of the scores of all classifiers were compared to select the best classifier and corresponding features. Kaplan-Meier (K-M) curves and log-rank tests were performed to analyze the significance of overall survival (OS) and PFS between patients predicted to have DCB (pDCB) and those predicted to have NDB (pNDB).

In the validation cohorts, the score was calculated for each patient using the same model as in the training cohort. Patients with melanoma in the validation cohorts were divided into pDCB and pNDB groups according to the same cut-off as the training cohort. The discriminatory ability of the DCB model in the validation cohorts was measured using the receiver operating characteristic (ROC) curve and the calculated value of area under the curve (AUC). K-M survival analysis was performed to analyze the significance of OS and PFS between the different groups.

### 2.3 Survival analysis

The impact of the DCB model, TMB and reported gene mutation signatures on survival outcomes in patients with melanoma treated with ICIs was explored. In addition, K-M survival analysis was performed for patients with lung cancer and ccRCC treated with ICIs as well as patients with melanoma without receiving ICIs therapy. The log-rank test was used to compare the survival curves. A *p* value < 0.05 was considered significant.

### 2.4 Genomic analysis associated with pDCB

TMB was determined as the number of non-synonymous mutations divided by the exome size (37). Gene mutations in pathways were analyzed with the previously reported gene lists (38–41). Patients with mutations in at least one gene in the DNA damage-repair pathway were defined as “altered DNA damage-repair pathway”. Mutation enrichment scores of antigen presentation, IFN-γ and ten classical oncogenic pathways were calculated for each sample using the single sample gene set enrichment analysis (ssGSEA) method (42).

### 2.5 Statistical analysis

All statistical analyses were performed using Python software version (3.10.1). For continuous and categorical variables, *t*-tests and Fisher’s exact tests were used, respectively. Statistical significance was set at *p*< 0.05.

## 3 Results

### 3.1 Construction of the DCB model to predict the response to the treatment of ICIs in melanoma

**Figure 1:**
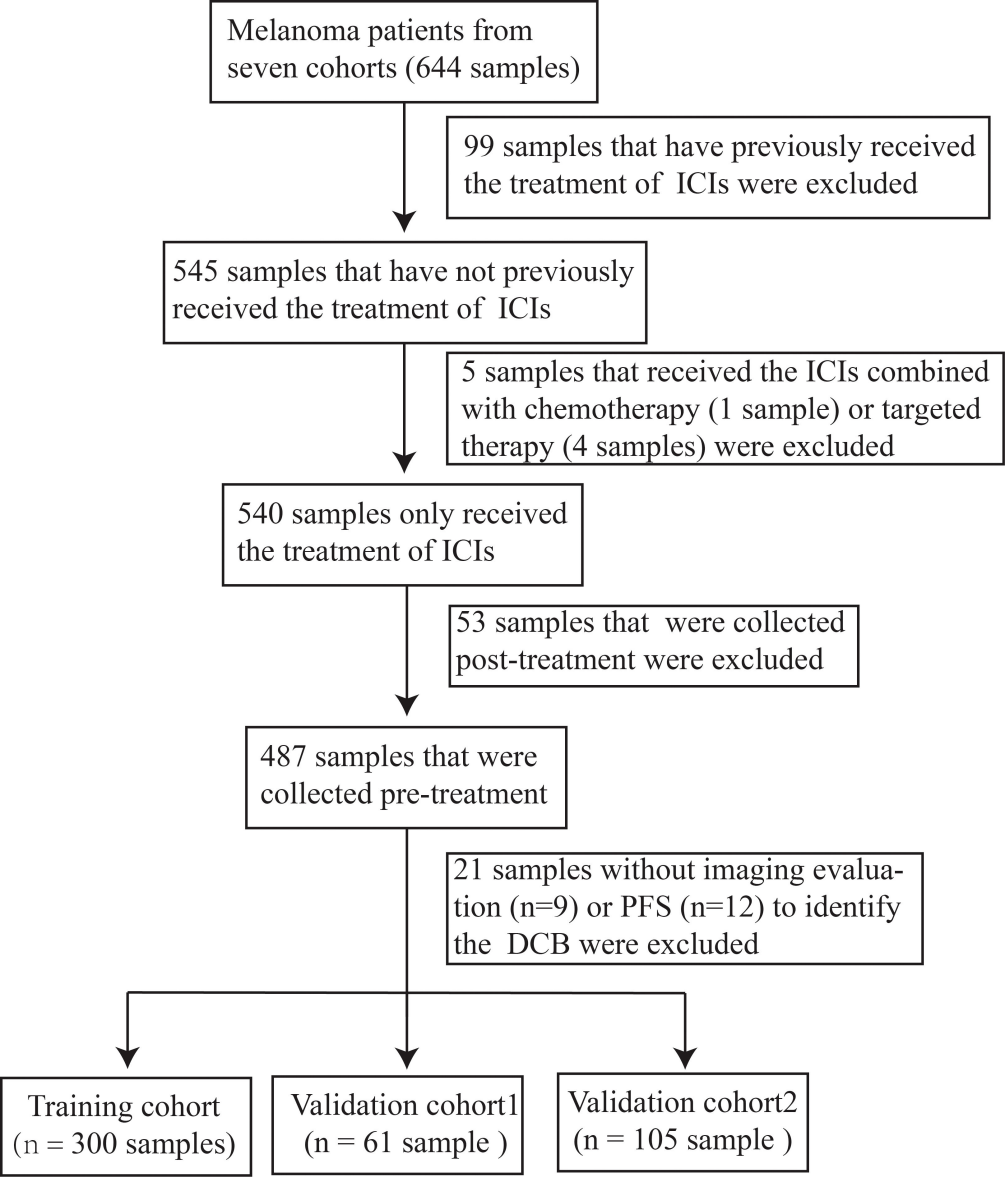
Sampling procedure for patients with melanoma treated with ICIs.

**Figure 2:**
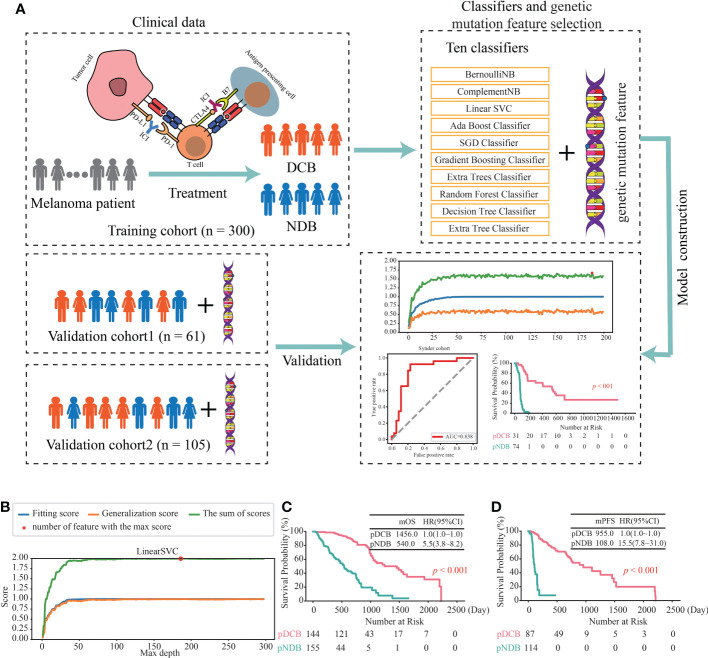
Construction of the DCB model for the treatment of ICIs in melanoma. **(A)** Workflow of the study. **(B)** The line chart of the f1 scores of fitting and generalization as well as the sum of the scores for Linear SVC in the training cohort. **(C, D)** Kaplan–Meier curves of OS **(C)** and PFS **(D)** comparing pDCB with pNDB from the DCB model in training cohort.

To investigate the association between the mutated genes and DCB for patients with melanoma receiving the therapy of ICIs, 644 samples from seven cohorts sequenced with WES were collected. After rigorous sample screening, 466 pre-treatment samples that received only the treatment of ICIs were obtained. Next, the 466 samples were divided into training cohort (n = 300), validation cohort1 (n = 61) and validation cohort2 (n = 105) (**Figure 1**). In the training cohort, ten classifiers (BernoulliNB, ComplementNB, Linear SVC, AdaBoost, SGD, Gradient Boosting, Extra Trees, Random Forest, Decision Tree and Extra Tree) were evaluated (**Figure 2A**). The best-performing hyperparameters were identified by the sum of the scores calculated from the 10-fold cross-validation. As shown in **Figures 2B**, **S1A–I**, the sum of the scores for Linear SVC with 186-gene mutation-based feature was the highest. The coefficient of each feature was obtained, and the score was calculated according to the formula in **Table S2**. Based on the above results, the Linear SVC with its corresponding features was selected as the DCB model. According to the scores calculated by the DCB model, patients with scores > 0 were classified as pDCB, and patients with scores ≤ 0 were classified as pNDB (**Table S3**). The results showed that the survival period of patients in the pDCB group was significantly longer than that of patients in the pNDB group (**Figures 2C, D**).

### 3.2 Validation of the DCB models in two independent cohorts

**Figure 3:**
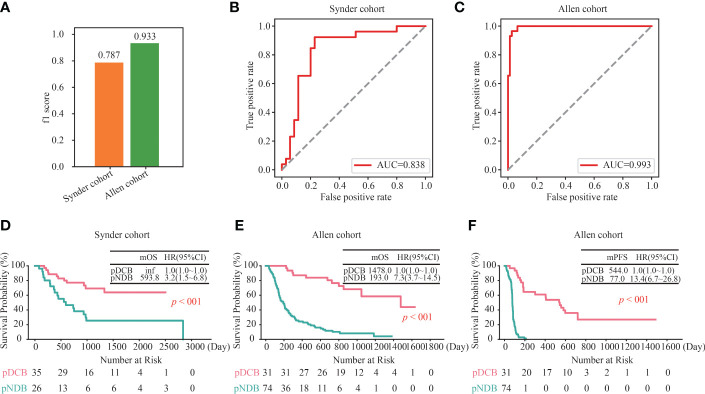
Validation of the DCB model in other independent cohorts. **(A)** f1 score of the DCB model in the Snyder and Allen cohorts. **(B, C)** ROC curves for the DCB model in the Snyder **(B)** and Allen **(C)** cohorts. **(D)** Kaplan–Meier curves of OS comparing pDCB with pNDB from the DCB model in Snyder cohort. **(E, F)** Kaplan–Meier curves of OS **(D)** and PFS **(E)** comparing pDCB with pNDB from the DCB model in Allen cohort.

To further assess the ability of the DCB model to predict the efficacy in patients with melanoma treated with ICIs, its performance in two independent validation cohorts was analyzed. As shown in **Figure 3A**, the f1 scores of the DCB model in the Snyder and Allen cohorts were 0.787 and 0.933, respectively. In addition, the AUC values of the DCB model in the Snyder and Allen cohorts were 0.838 and 0.993, respectively (**Figures 3B, C** and **Table S3**). These results indicated that the DCB models can effectively predict patient outcomes. The predictive effect of the DCB model on prognosis in the two independent validation cohorts was determined. The results showed that patients with pDCB had longer median OS (mOS) and median PFS (mPFS) than those with pNDB (**Figures 3D**–**F**). Furthermore, 282 patients with melanoma who did not receive ICIs treatment were included to evaluate the predictive effect of the DCB model. As shown in **Figure S2A** and **Table S4**, there was no significant difference between the survival curves of pDCB and pNDB, indicating that the model was ICIs-specific. To expand the application of this model, its predictive ability in patients with lung cancer and ccRCC treated with ICIs was analyzed. We found no significant difference between the pDCB and pNDB groups (**Figures S2B, C** and **Table S5**). This may be due to the heterogeneity among tumors. As shown in **Figure S2D**, the mutation frequency of the top 10 gene in the model varies greatly in melanoma, lung cancer and ccRCC.

### 3.3 The predictive ability of the DCB model was superior to TMB and other reported genetic mutation signatures

**Figure 4:**
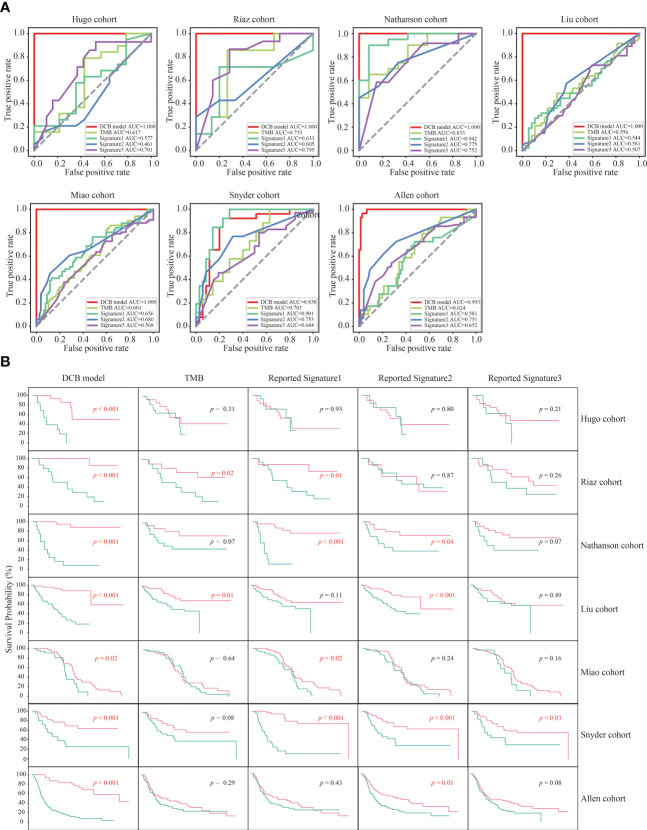
The predictive ability of the DCB model was superior to TMB and other reported genetic mutation signatures. **(A)** ROC curves of the DCB model, TMB and reported genetic mutation signature 1, 2 and 3 in the seven melanoma cohorts. **(B)** Kaplan–Meier curves of OS comparing pDCB with pNDB from the DCB model, TMB and reported genetic mutation signature 1, 2 and 3 in the seven melanoma cohorts.

Previous studies have revealed that TMB and three reported genetic mutation signatures (Signature1 from the study of Lu et al., Signature2 from the study of Jiang et al., and Signature3 from the study of Long et al.) can serve as biomarkers to effectively distinguish the responders among patients with melanoma receiving the treatment of ICIs (14, 16, 24–26). Next, we compared the predictive effect of the DCB model, TMB and the reported genetic mutation signatures, and found that the f1 score and AUC value of the DCB model was higher than that of the TMB and three reported genetic mutation signatures in the six cohorts (Hugo, Riaz, Nathanson, Liu, Miao and Allen cohorts); only in the Snyder cohort, the f1 score and AUC value of the reported genetic mutation signature 1 was slightly higher than that of the DCB model (**Figures S3**, **4A**). For TMB and the reported genetic mutation signature 1, there were one (Nathanson cohort) and two cohorts (Nathanson and Snyder cohorts) with AUC values above 0.8, respectively. For the reported genetic mutation signature 2 and 3, none of the cohorts had AUC values exceeding 0.8. The maximum AUC values for the reported genetic mutation signatures 2 and 3 were 0.775 (Nathanson cohort) and 0.795 (Riaz cohort), respectively. Furthermore, their predictive effects on prognosis were evaluated. For the DCB model, there were significant differences between pDCB and pNDB across all seven cohorts. However, for TMB and the reported genetic mutation signature 1, 2 and 3, the survival curves between pDCB and pNDB were significantly different in two cohorts (Riaz and Liu cohorts), four cohorts (Riaz, Nathanson, Miao and Snyder cohorts), four cohorts (Nathanson, Liu, Snyder and Allen cohorts) and one cohort (Snyder cohorts), respectively (**Figure 4B**). These results demonstrated that the DCB model showed greater predictive power than TMB and other reported genetic mutation signatures in patients with melanoma treated with ICIs.

### 3.4 Distinctive genomic patterns associated with the DCB model.

**Figure 5:**
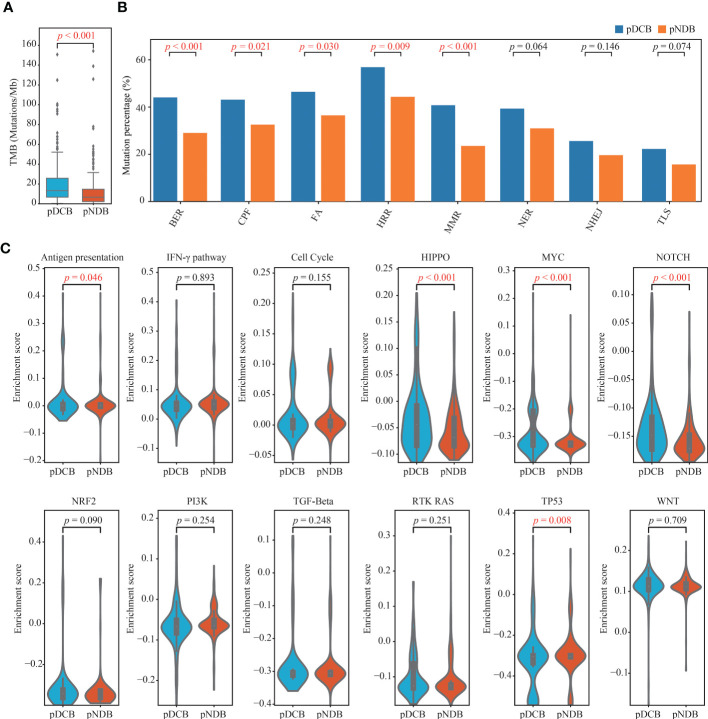
Distinctive genomic patterns associated with the DCB model. **(A)** Comparison of TMB between patients with pDCB and pNDB. **(B)** Barplots of DNA damage repair pathways mutation percentage between pDCB and pNDB. **(C)** Comparison of the enrichment scores from antigen presentation, IFN-γ and ten oncogenic pathways between pDCB and pNDB.

To explore the underlying factors by which the DCB model can effectively predict the outcome of immunotherapy, the tumor immunogenicity between the pDCB and pNDB groups was analyzed. As shown in **Figures 5A**, **B**, TMB and most DNA damage pathway mutation percentages were significantly higher in the pDCB group than in the pNDB group, which has been reported to be associated with the clinical benefit of ICIs. Genes that were significantly associated with pDCB or pNDB were TTN (Pearson-r = 0.3007) and XIRP2 (Pearson-r = 0.3290). To further characterize the mutation process between the pDCB and pNDB groups, the enrichment scores for antigen presentation, IFN-γ and ten classical oncogenic pathways were calculated. The scores for the Hippo, MYC and Notch pathways were significantly higher in the pDCB group, whereas the antigen presentation and TP53 pathways were enriched in the pNDB group (**Figure 5C**). Mutations in the Hippo and Notch pathways have been reported to improve the clinical benefits of patients treated with ICIs, while mutations in the antigen presentation pathway leaded to tolerance to ICIs treatment (43–45).

## 4 Discussion

The treatment landscape of multiple cancers including melanoma has been revolutionized by ICIs, such as anti-PD-L1, anti-PD-1 and anti-CTLA4. However, selecting responders to the treatment of ICIs is the leading challenge in this field. In our study, classifiers were systematically screened to predict the response to the treatment of ICIs in melanoma, and a robust DCB model was developed based on the Linear SVC with 186-gene mutation-based feature. The AUC value of the ROC curves and the significant difference in the survival curves from the pDCB and pNDB groups showed that our DCB model had high performance in both the training and validation cohorts. Compared with the identified biomarker TMB and other reported genetic mutation signatures, our DCB model had the highest precision and accuracy in predicting response to the treatment of ICIs. Furthermore, higher immunogenicity was associated with the melanoma patients with pDCB.

Multiple studies have identified biomarkers to predict the response in patients with melanoma treated with ICIs (14, 30, 35, 46). However, their sensitivities and accuracies were limited. For example, TMB-high patients with loss of heterozygosity at the human leukocyte antigen or mutations in antigen presentation, and interferon-receptor signaling pathways were still resistant to the treatment of ICIs (45, 47–49). In this study, a DCB model based on 186-gene mutation-based feature was constructed, and performed better than the identified biomarker TMB. Furthermore, the genetic mutation feature used to construct the DCB model can be designed as a customized targeted panel, and sequencing can be performed to distinguish responders, which is more convenient and less expensive.

Three genetic-mutation feature based signatures have been reported to predict the clinical benefit for patients with melanoma treated with ICIs (24–26). However, these signatures were constructed without rigorous screening of the samples, resulting in the inclusion of post-treatment samples. In this study, the samples were strictly screened (**Figure 1**). Only pre-treatment samples from patients who had not previously received the treatment of ICIs were retained, so that the DCB model had predictive significance. As shown in **Figure 4A** and **4B**, the predictive power of our DCB model is more effective and robust than other reported genetic-mutation feature based signatures for patients with melanoma. Previous studies have revealed that clinical characteristics may affect the outcome of ICIs (50, 51). In our model, we found that adding gender, age and clinical stage to the model did not significantly improve its prediction power (**Figure S4**).

Furthermore, the genomic features of melanoma patients with pDCB were investigated. Mutation in DNA damage repair pathways and TMB were associated with the pDCB patients. Mutations in DNA damage repair pathways reduce the genomic stability. Mutated genes cannot be repaired in a timely and effective manner, leading to the accumulation of mutations (38). High TMB increases the presentation of immune neoantigens and enhances tumor immunogenicity, thereby inducing effective anti-tumor immune responses. These results may explain why melanoma patients with pDCB were more likely to benefit from the treatment of ICIs.

It is noteworthy that there were several limitations in our study. First, although we have elucidated the genomic features of the DCB model, we still need to explore the mechanism by which mutations in each gene affect the treatment of ICIs in patients with melanoma. Second, since both the training and validation cohorts in our study were from retrospective studies, the obtained genetic mutation features may be subject to cohort selection bias. Therefore, prospective studies are required to validate this DCB model.

In conclusion, our study systematically screened the suitable classifier based on genetic mutation features to construct the DCB model that can effectively distinguish patients with melanoma who might benefit from the treatment of ICIs. The predictive power of our DCB model is more effective and robust than that of the reported genomic biomarker TMB and genetic mutation feature-based signatures. The genomic features between pDCB and pNDB groups were also explored. Overall, the constructed DCB model warrants validation by future prospective studies and may help guide clinical decision-making.

## Data availability statement

The original contributions presented in the study are included in the article/**Supplementary Material**. Further inquiries can be directed to the corresponding authors.

## Author contributions

Conceptualized the manuscript, HL, LX and X-JL. Developed methodology, CP and HT. Analyzed and interpreted the data, CP, HT and WW. Wrote the manuscript, CP and HT. Revised the manuscript, WW, DW, HL, LX. and X-JL. All authors contributed to the article and approved the submitted version.

## Supplementary material

Supplementary Figure 1 The predictive effect of 9 classifiers in the training cohort. **(A)** - (I)The line charts of the f1 scores of fitting and generalization as well as the sum of the scores for BernoulliNB **(A)**, ComplementNB **(B)**, AdaBoost Classifier **(C)**, SGD Classifier **(D)**, Gradient Boosting Classifier **(E)**, Extra Trees Classifier **(F)**, Random Forest Classifier **(G)**, Decision Tree Classifier **(H)** and Extra Tree Classifier **(I)** in the training cohort.

Supplementary Figure 2 Survival analysis of the DCB model in patients with melanoma without ICIs treatment and patients with lung cancer and ccRCC treated with ICIs. **(A)** Kaplan–Meier curves of OS comparing pDCB with pNDB in the patients with melanoma without ICIs treatment. **(B)** Kaplan–Meier curves of PFS comparing pDCB with pNDB in the patients with lung cancer treated with ICIs. **(C)** Kaplan–Meier curves of OS comparing pDCB with pNDB in the patients with ccRCC treated with ICIs. **(D)** The mutation frequency of top 10 gene from the DCB model in melanoma, lung cancer and ccRCC.

Supplementary Figure 3 The f1 scores of the DCB model, TMB and reported genetic mutation signature 1, 2 and 3 in the seven melanoma cohorts.

Supplementary Figure 4 The f1 scores of 186 features, 186 features plus sex and age as well as 186 features plus sex and stage in the Snyder and Allen cohorts.
