# Supplementary figures and images for: An enhanced genetic mutation-based model for predicting the efficacy of immune checkpoint inhibitors in patients with melanoma
Source: Front Oncol. 2023 Jan 17;12:1077477. doi: 10.3389/fonc.2022.1077477 (PMC9887306; doi:10.3389/fonc.2022.1077477)

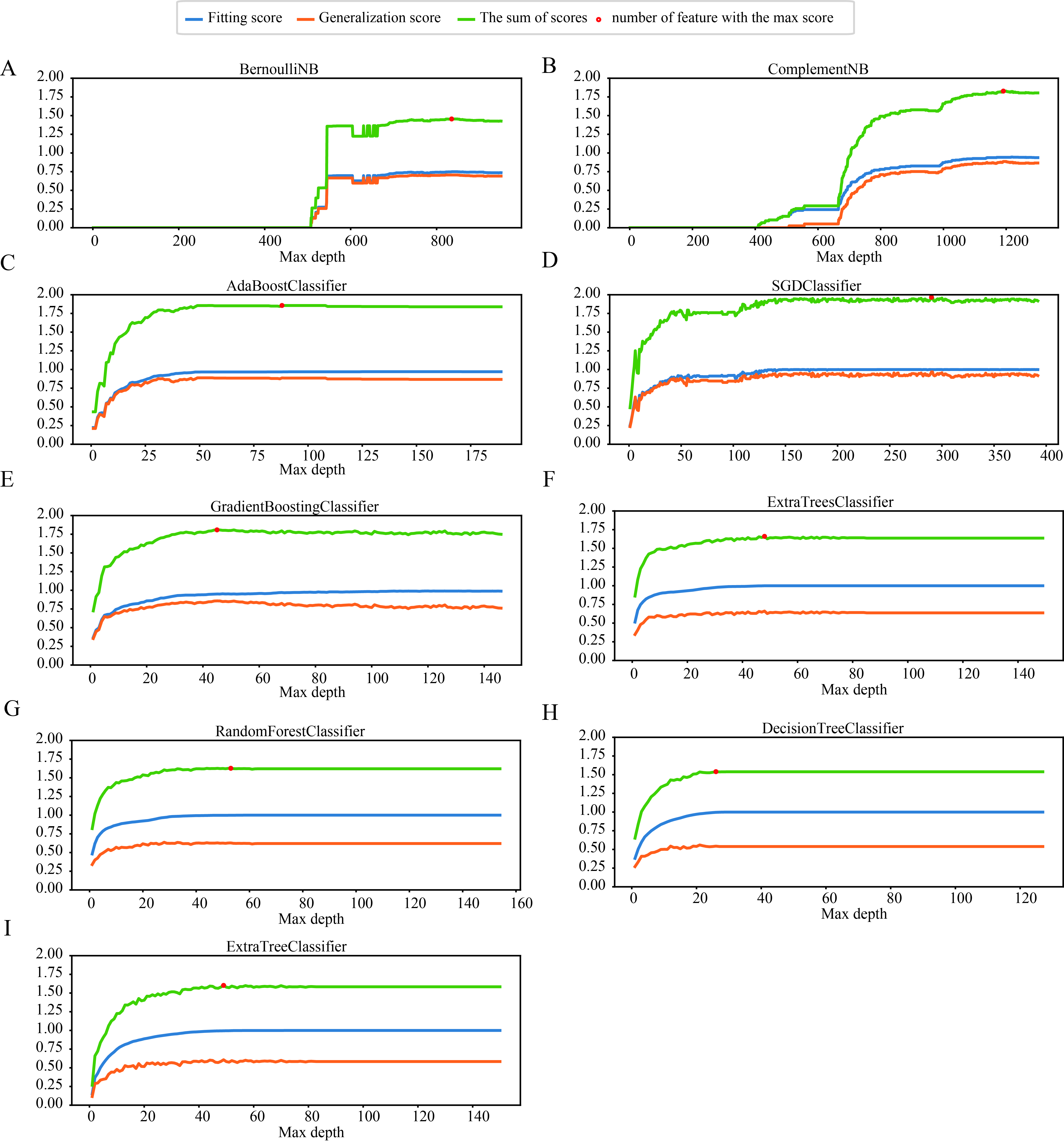

Supplement: Supplementary Figure 1 — The predictive effect of 9 classifiers in the training cohort. (A) - (I)The line charts of the f1 scores of fitting and generalization as well as the sum of the scores for BernoulliNB (A), ComplementNB (B), AdaBoost Classifier (C), SGD Classifier (D), Gradient Boosting Classifier (E), Extra Trees Classifier (F), Random Forest Classifier (G), Decision Tree Classifier (H) and Extra Tree Classifier (I) in the training cohort. [file Image_1.jpeg]

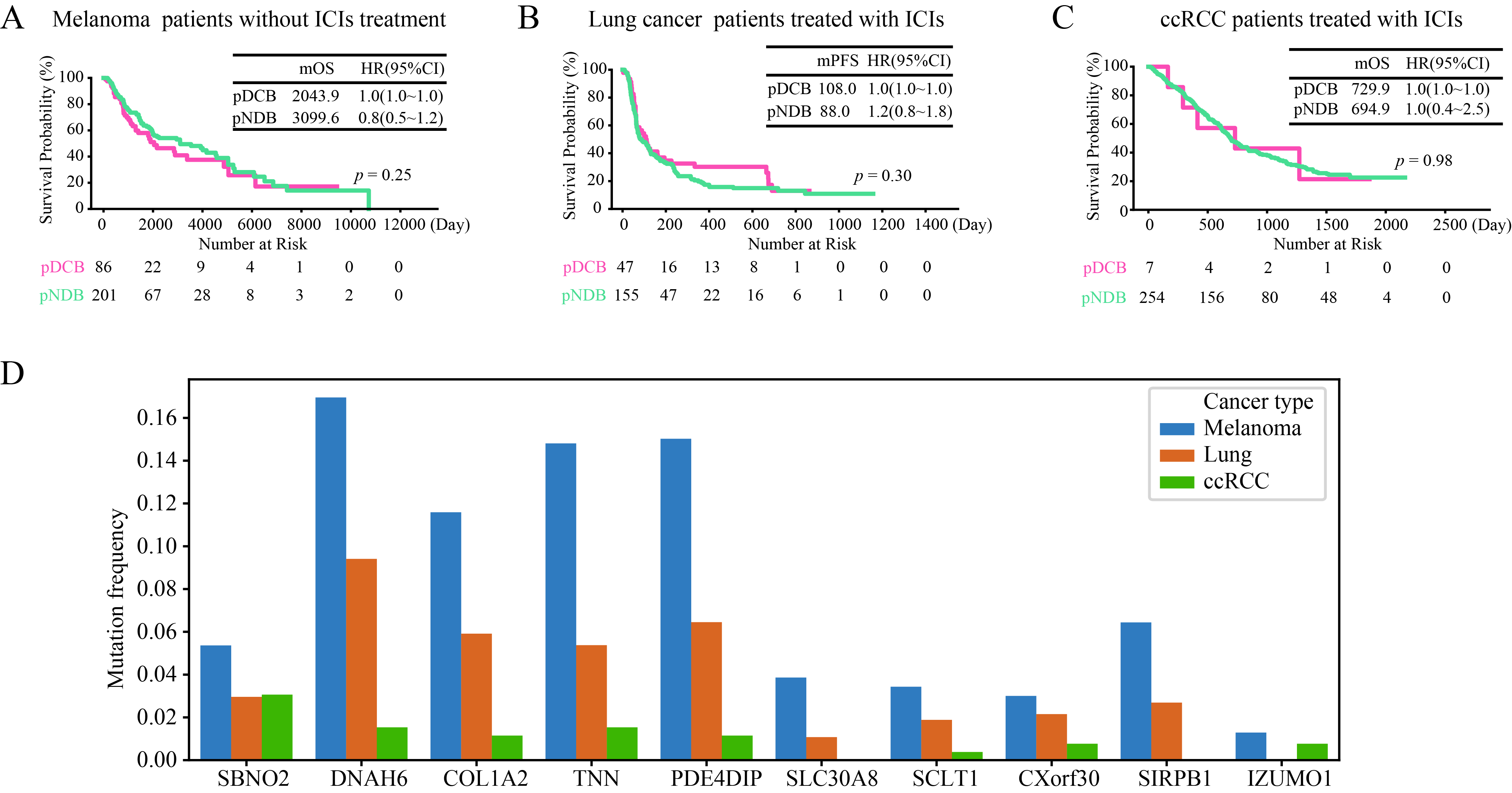

Supplement: Supplementary Figure 2 — Survival analysis of the DCB model in patients with melanoma without ICIs treatment and patients with lung cancer and ccRCC treated with ICIs. (A) Kaplan–Meier curves of OS comparing pDCB with pNDB in the patients with melanoma without ICIs treatment. (B) Kaplan–Meier curves of PFS comparing pDCB with pNDB in the patients with lung cancer treated with ICIs. (C) Kaplan–Meier curves of OS comparing pDCB with pNDB in the patients with ccRCC treated with ICIs. (D) The mutation frequency of top 10 gene from the DCB model in melanoma, lung cancer and ccRCC. [file Image_2.jpg]

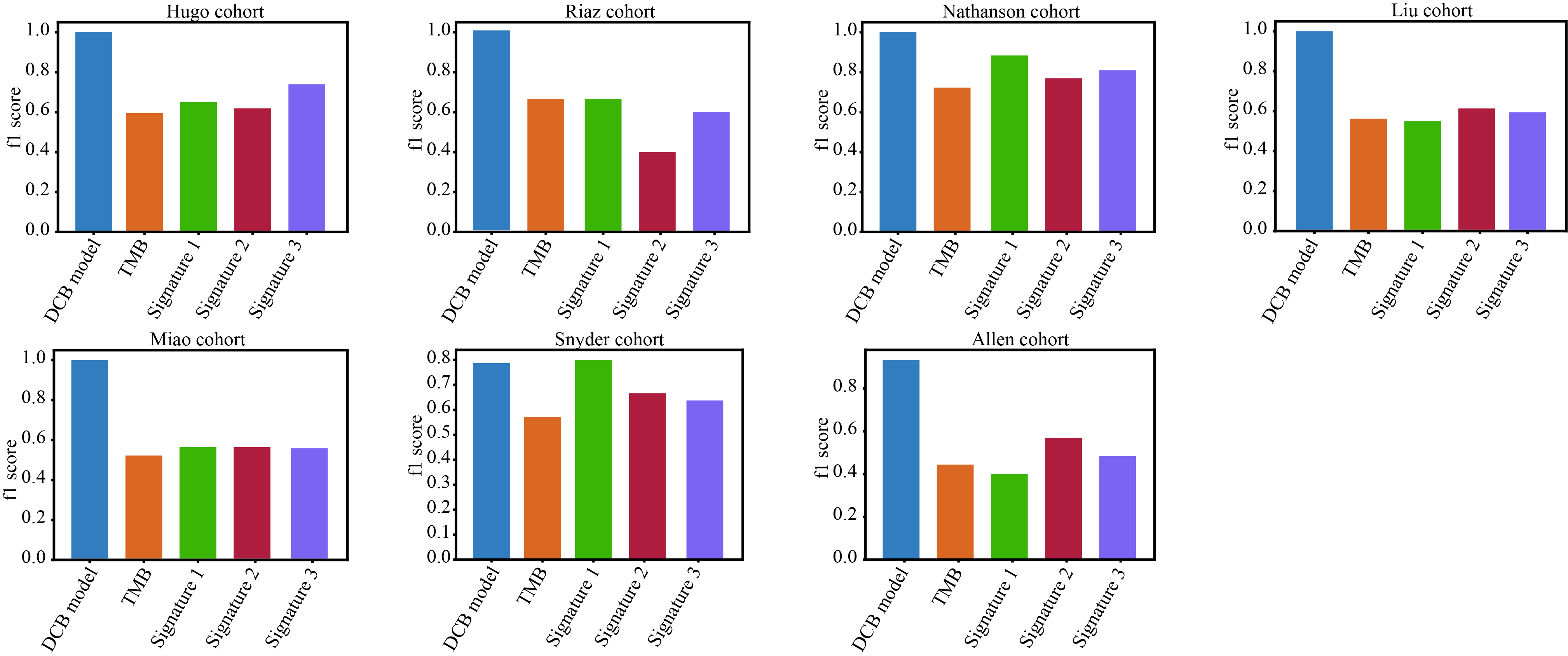

Supplement: Supplementary Figure 3 — The f1 scores of the DCB model, TMB and reported genetic mutation signature 1, 2 and 3 in the seven melanoma cohorts. [file Image_3.jpeg]

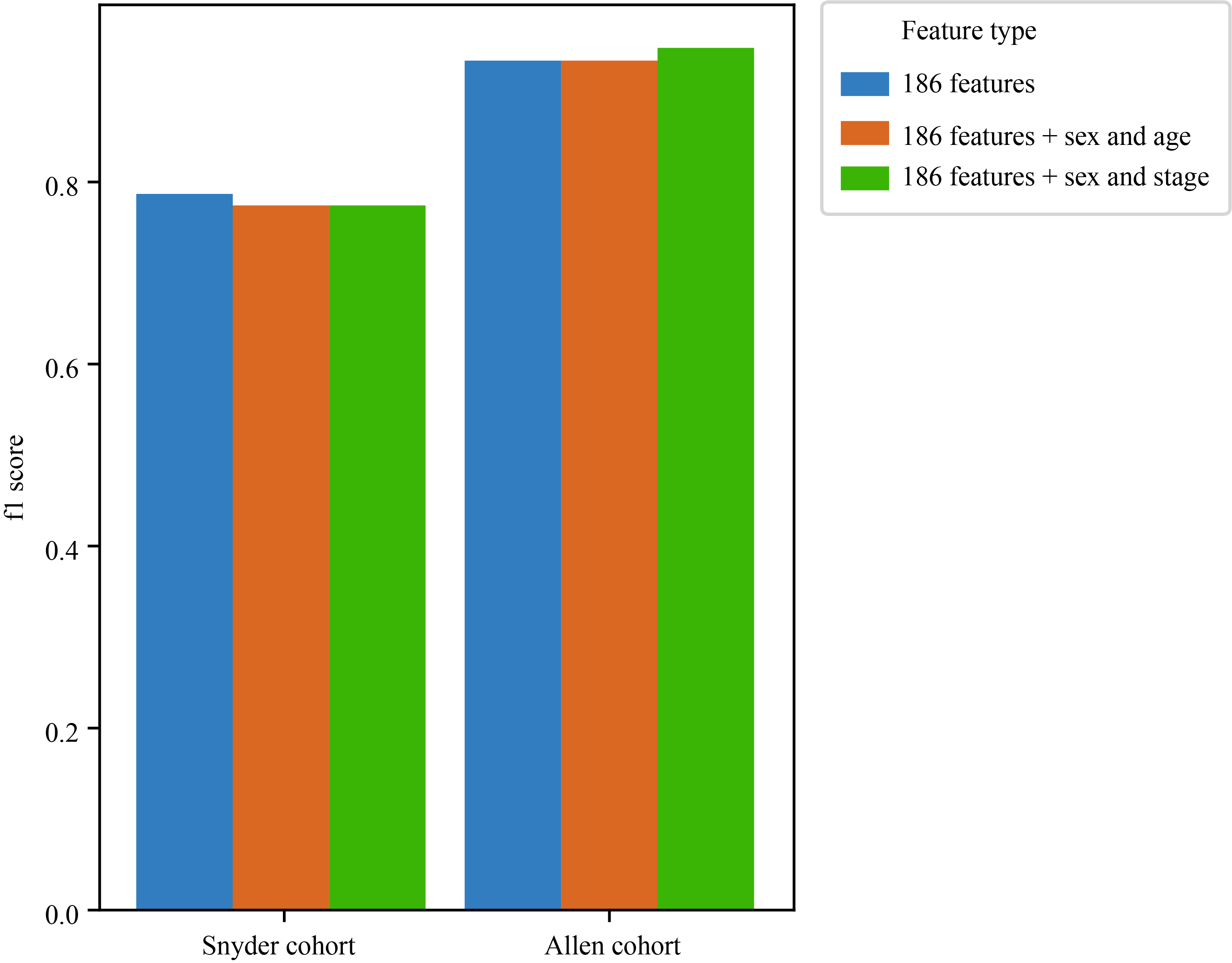

Supplement: Supplementary Figure 4 — The f1 scores of 186 features, 186 features plus sex and age as well as 186 features plus sex and stage in the Snyder and Allen cohorts. [file Image_4.jpeg]
